# Hybrid Version of the Kedem–Katchalsky–Peusner Equations for Diffusive and Electrical Transport Processes in Membrane

**DOI:** 10.3390/membranes15010036

**Published:** 2025-01-20

**Authors:** Andrzej Ślęzak, Sławomir M. Grzegorczyn

**Affiliations:** 1 Department of Health Sciences and Physiotherapy, Collegium Medicum, Jan Dlugosz University, 13/15 Armia Krajowa Al., 42-200 Częstochowa, Poland; 2 Department of Biophysics, Faculty of Medical Sciences in Zabrze, Medical University of Silesia, 19 H. Jordan Str., 41-808 Zabrze, Poland

**Keywords:** membrane transport, Kedem–Katchalsky–Peusner equations, polymeric membrane, Peusner transport coefficients, internal energy conversion, *S*-entropy

## Abstract

One of the most important formalisms used to describe membrane transport is Onsager–Peusner thermodynamics (TOP). Within the TOP framework, a procedure has been developed for the transformation of the Kedem–Katchalsky (K–K) equations for the transport of binary electrolytic solutions across a membrane into the Kedem–Katchalsky–Peusner (K–K–P) equations. The membrane system with an Ultra Flo 145 Dialyser membrane used for hemodialysis and aqueous NaCl solutions was used as experimental setup. The H version of K–K–P formalism for binary electrolyte solutions was used to evaluate theoretical coefficients characterizing fluxes of energies and efficiencies for membrane transport processes. The coupling coefficients of membrane processes and the dissipative energy flux were calculated on the basis of the Peusner coefficients obtained from transformation of K–K coefficients. The knowledge of dissipative energy flux, which is a function of thermodynamic forces, allows for the determination of the energy conversions during transport processes in a membrane system. In addition, a frictional interpretation of the obtained coefficients is presented.

## 1. Introduction

Membrane transport as one of the basic nonequilibrium processes is observed in various types of life-supporting biological and physicochemical systems and in applied technological processes [1]. The typical examples of such processes are membrane dressings to promote the healing of chronic wounds, the cellular and tissue systems of living organisms, controlled drug release systems, and various systems with energy conversion [2,3,4]. In the aforementioned systems, polymeric membranes with a porous, capillary, or mosaic structure and biocompatible composition, made of bacterial cellulose, polyvinyl chloride, or cellulose acetate, mimicking to some extent the biological membrane, provide a selective barrier to ensure their desired functionality [5,6].

A convenient and widely used tool for the study of membrane transport processes is the network thermodynamics (NT) in Oster, Perelson and Katchalsky version [7], and in the Peusner version (Peusner NT) [8,9,10]. The latter uses Onsager’s nonequilibrium thermodynamics and the symbolism and laws of analog electric circuit theory [11,12,13]. One of the significant scientific achievements of L. Peusner is the development of Kedem and Caplan’s idea of the degree of coupling of thermodynamic processes by introducing phenomenological coefficients, symmetric (L and R) and hybrid (H and P), to evaluate the efficiency of energy conversion [9,10].

In recent years, the importance of a formalism that combines the Kedem–Katchalsky equations with the research tools of Peusner’s network thermodynamics has been growing [9,10,14]. The starting point for these considerations is the energy dissipation function, which is the product of entropy production and absolute temperature and is a measure of the dissipation of energy in the system. In turn, as a measure of the irreversibility of the transport processes of mass and charge, entropy production can be used. Energy dissipation is also treated as a basis for the derivation of the Kedem–Katchalsky equations of membrane transport and the equations for the conversion of internal energy [12,13,14,15,16].

**Figure 1:**
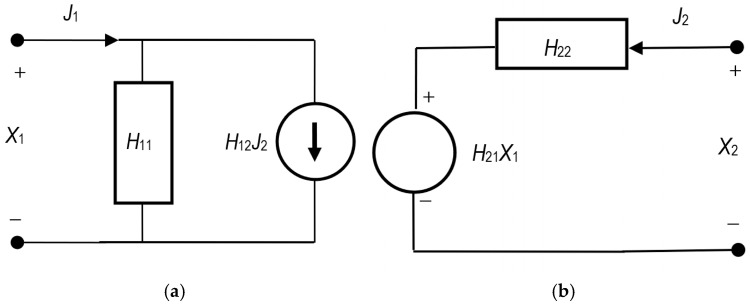
Two-port *H* diagrams of Equations (1) (**a**) and (2) (**b**), in which forces ($X_{1},\;X_{2}$) controlling flows ($J_{1},\;J_{2}$) are arranged in parallel with conductivities ($H_{11}$, $H_{22}$) [9,10].

According to Peusner’s idea, transducers for binary solutions are marked as L, R, H, and P [9,10]. Their main components are two controllable sources (of thermodynamic force or flow) and two dissipative elements (conductance or resistance). In this paper, we will only consider the H version of Peusner’s equations. The model schemes for the H version of Peusner network thermodynamics are shown in Figure 1.

The *H* version of phenomenological equations for linear two-port can be presented as:

(1) $$J_{1}=H_{11}X_{1}+H_{12}J_{2}$$

(2) $$X_{2}=H_{21}X_{1}+H_{22}J_{2}$$

These equations are hybrid in the sense that they combine forces and fluxes, but lead to a useful two-port representation. As in the case of the L and R versions, for the H version, no rigorous fulfillment of reciprocity relations is assumed [13,17,18]. Equations (1) and (2) give practical interpretations of the coefficients of $H_{ij}$ (*i*, *j* ∈ {1, 2}): $H_{11}={\left(J_{1}/X_{1}\right)}_{J_{2}=0}$, $H_{12}={\left(J_{1}/J_{2}\right)}_{X_{1}=0}$, $H_{21}={\left(X_{2}/X_{1}\right)}_{J_{2}=0}$, and $H_{22}={\left(X_{2}/J_{2}\right)}_{X_{1}=0}$, for open (zero flow) and short-circuit (zero force) conditions. The coefficients in the hybrid notation combine directly coupled thermodynamic forces and fluxes (*H*_11_ and *H*_22_) and determine the relationship between fluxes (*H*_12_) and forces (*H*_21_) in the processes of the membrane system. In this approach to non-equilibrium thermodynamics, it is possible, through coefficients, not only to express the cross-relations between thermodynamic forces and fluxes, but also to determine direct connections between the observed fluxes.

The H-circuit schemes shown in Figure 1a,b represent a two-port flow with hybrid element $H_{11}$ connected in parallel to source $J_{1}=H_{12}J_{2}$ (a) and with hybrid element $H_{22}$ connected in series to source $J_{2}=H_{21}J_{1}$ (b). The total flow in case (a) is represented by Equation (1) and in case (b) by Equation (2). In the hybrid representation, $X_{1}$ and $J_{2}$ are the independent variables. The symmetrical coefficients in the hybrid approach (*H*_11_ and *H*_22_) express the respective electrical (or “diffusion”) conductivities in the hybrid system, while the asymmetric dimensionless coefficients express the relations between voltage sources (thermodynamic forces) or current densities (thermodynamic fluxes), respectively.

Previous works have presented procedures for analyzing the membrane transport of binary homogeneous electrolyte solutions using the L and R versions of the Kedem–Katchalsky–Peusner equations [19,20,21]. The procedure for converting chemical energy to free energy has also been presented. The beginning of this procedure was the calculation of energy dissipation by means of the *L* or *R* version of the K–K–P equations. Thermodynamic forces (differences in osmotic pressure, electromotive force, etc.) and fluxes of solute and ionic current were used in these procedures.

The purpose of this work is to elaborate on a procedure for the hybrid conversion of K–K equations for binary electrolyte solutions to K–K–P equations using the formalism developed on the basis of PNT. The transformation of internal to free energy conversion in a membrane system containing aqueous electrolyte solutions with a concentration and an electric field superimposed on the membrane was developed using the *H* version of the K–K–P equations. The work is organized as follows.

The paper begins with an introduction, and the Section 2 presents the procedure for deriving the *H* versions of the K–K–P equations describing the transport of homogeneous electrolyte solutions through the membrane. In addition, the Section 2 also contains a derivation of the coupling coefficients $h_{ij}$ and $Q_{H}$ and the energy conversion efficiency coefficient ${\left(e_{H}\right)}_{max}$. The equations for the energy dissipation function ${\left(\Phi_{S}\right)}_{H}$ are also provided in this section. The obtained equations were used for the calculations: ${\left(\Phi_{S}\right)}_{H}$ = *f*($\Delta \pi_{s}/C_{s}$, *I*), ${\left(\Phi_{F}\right)}_{H}$ = *f*($\Delta \pi_{s}/C_{s}$, *I*), and ${\left(\Phi_{U}\right)}_{H}$ = *f*($\Delta \pi_{s}/C_{s}$, *I*) based on the characteristics $H_{ij}$ = *f*($\Delta \pi_{s}/C_{s}$, *I*) and $H_{ij}$ = *f*($\Delta \pi_{s}/C_{s}$, *I*), for Ultra Flo 145 Dialyzer membrane and aqueous NaCl solutions. The values of the coupling parameter and energy conversion efficiency coefficient ($e_{H}{)}_{max}$ were used to evaluate the electrochemical energy conversion. The Section 3 contains the results obtained from measurement and calculation and their discussion, while the Section 4 includes the summary and conclusions.

## 2. Materials and Methods

### 2.1. Membrane System

**Figure 2:**
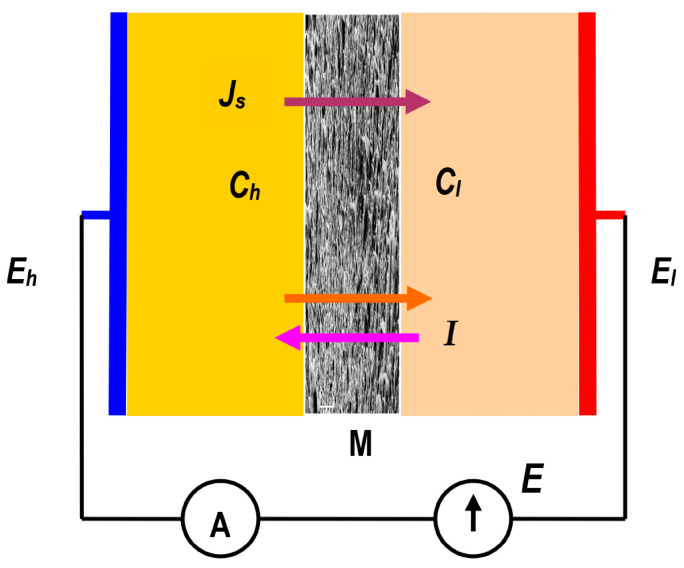
The model of a membrane system: M—Ultra Flo 145 Dialyzer membrane, $C_{h}$ and $C_{l}$—NaCl concentrations ($C_{h}$ > $C_{l}$), $J_{s}$—solute flux, I—electric ionic current, $E_{h}$ and $E_{l}$—electrode potentials.

A scheme of the system used to study the membrane transport is shown in Figure 2. The system consisted of two chambers (*l*, *h*) made of Plexiglas separated by a membrane (M), placed in a vertical plane. One of the chambers was connected to a calibrated pipette and the other to a solution reservoir. At baseline, chamber *l* was filled with an aqueous NaCl solution with concentration $C_{l}$ = const., while chamber *h* was filled with concentration $C_{h}$ > $C_{l}$. The density of solutions with $C_{h}$ and $C_{l}$ concentrations fulfilled the condition $\rho_{h}$ > $\rho_{l}$ = const. The solutions separated by the membrane were mechanically stirred with frequency 500 rpm. Ag/AgCl electrodes in the form of a flat disk were placed in each chamber.

The electrodes had equal thickness and equal surface area. As is well known, there are two driving forces in such a system: the osmotic pressure difference ($\Delta \pi_{s}$), generating the solute flux $(J_{s}$), and the electromotive force (E), generating the electric ionic current (I). The voltage was applied to the electrodes using a suitable DC power supply.

The experiments were conducted in a chamber with a stabilized temperature (*T* = 295 K); the metal chamber was also insulated and grounded to ensure the elimination of electrical interference from external sources. The membrane used for the experiments was an Ultra Flo 145 Dialyzer regenerated cellulose membrane (Artificial Organs Division, Travenol Laboratories S.A., Brussels, Belgium) cut in the form of a disk from a hemodialysis hose, which was a part of the “coiled artificial kidney” used in medicine in the second half of the 20th century [22]. The scan image of the Ultra Flo 145 dialyzer membrane presented in a previous paper [21] shows the membrane as a compact structure with visible cellulose fiber residues. According to Kedem–Katchalsky formalism, the transport parameters of a membrane are determined by six coefficients: diffusion permeability ($\omega_{s}$), transference number ($\tau_{s}$), and conductance (κ).

### 2.2. H Version of the Kedem–Katchalsky–Peusner Equations

The H versions of the Kedem–Katchalsky–Peusner equations for homogeneous electrolyte solutions are obtained by an appropriate transformation of the classical Kedem–Katchalsky equations for homogeneous electrolyte solutions with the assumption $J_{v}$ = 0 [10,14]:

(3) $$J_{s}=\omega_{s}\Delta \pi_{s}+\frac{\tau_{s}}{z_{s}F}I$$

(4) $$I=\frac{\kappa \tau_{s}}{z_{s}F}\frac{\Delta \pi_{s}}{C_{s}}+\kappa E$$

where $\omega_{s}$—coefficient of diffusion permeability, $J_{s}$—solute flux, *I*—electric ionic current, $\Delta \pi_{s}$ = RTΔC—osmotic pressure difference, RT—the product of the gas constant and the absolute temperature, ΔC = $C_{h}-C_{l}$ ($C_{h}>C_{l}$)—difference of concentrations on the mem-brane, $C_{s}=\left(C_{h}-C_{l}\right){\left(lnC_{h}{C_{l}}^{-1}\right)}^{-1}$ = $\Delta \pi_{s}{\left(RTlnC_{h}{C_{l}}^{-1}\right)}^{-1}$ ≈ 0.5$\left(C_{h}+C_{l}\right)$—average solute concentration in the membrane, $\frac{\Delta \pi_{s}}{C_{s}}=\ln\left(C_{h}{C_{l}}^{-1}\right)$, E—is the potential difference (voltage) across membrane, $\tau_{s}$—transference number, F—Faraday constant, κ—conductance coefficient, and $z_{s}$—valence of *s*-ion.

The phenomenological coefficients appearing in Equations (3) and (4) are defined by the following expressions [9]:

(5) $$\omega_{s}={\left(\frac{J_{s}}{\Delta \pi_{s}}\right)}_{\;I=0}$$

(6) $$\kappa ={\left(\frac{I}{E}\right)}_{\Delta \pi_{s}=0}$$

(7) $$\tau_{s}=\frac{z_{s}F}{C_{s}}{\left(\frac{J_{s}}{I}\right)}_{\Delta \pi_{s}=0}$$

Using the procedure proposed by Kedem and Katchalsky, the coefficients $\omega_{s}$, κ, and $\tau_{s}$ can be expressed by the membrane friction coefficients [14]:

(8) $$\omega_{s}=\frac{K\;\vartheta }{\Delta x\left(f_{sw}+f_{sm}\right)}=\frac{C_{s}{\varphi_{w}}^{2}\vartheta }{C\Delta xf_{s}^{0w}}$$

(9) $$\kappa =\frac{F^{2}X\;\vartheta }{f_{1w}\Delta x}=\frac{\vartheta XF^{2}}{f_{1}^{0w}\Delta x}$$

(10) $$\tau_{2}={\left(\frac{C_{s}\varphi_{w}}{X}\right)}^{2}\frac{f_{1w}}{f_{2w}}$$

where *K*—distribution coefficient for salt between aqueous solution and membrane, Δ*x*—thickness of the membrane, $f_{iw}$—friction coefficient between *i*-th ion and water molecules, $f_{im}$—friction coefficient between *i*-th ion and membrane, *X*—fixed charges concentration in the membrane matrix, (indexes: 1—for counterion, 2—for coion), $\varphi_{w}$—volume of water in membrane, *ϑ*—winding coefficient of channels in membrane, $C_{s}$—average solute concentration in the membrane, $f_{iw}^{0}$—friction coefficient in free solution, and *i* = 1, 2.

Equations (8)–(10) represent a frictional interpretation of the coefficients $\omega_{s}$, κ, and $\tau_{s}$. Equations (8) and (9) show that the values of the coefficients $\omega_{s}$ and κ decrease as the thickness of the membrane and the friction of the solute with water and membrane increase. In turn, an increase in the value of the K coefficient leads to an increase in the $\omega_{s}$ coefficient. The coefficient ϑ should be taken into account when the lengths of the channels inside the membrane are longer than the macroscopic thickness of the membrane Δx. The actual dimension of the membrane channels is given by the ratio Δx/ϑ, in which  ϑ < 1. The value of the $\tau_{s}$ coefficient increases with the square of $C_{s}\varphi_{w}$ and decreases with the square of X. Moreover, the value of the $\tau_{s}$ coefficient is directly proportional to $f_{1w}/f_{2w}$.

Transforming Equations (3) and (4) with Peusner’s network thermodynamics method, we get the H version of Equations (3) and (4):

(11) $$J_{s}=H_{11}\frac{\Delta \pi_{s}}{C_{s}}+H_{12}I$$

(12) $$E=H_{21}\frac{\Delta \pi_{s}}{C_{s}}+H_{22}I$$

where:

(13) $$H_{11}={\left(\frac{J_{s}}{\Delta \pi_{s}/C_{s}}\right)}_{I=0}=C_{s}\omega_{s}$$

(14) $$H_{12}={\left(\frac{J_{s}}{I}\right)}_{\Delta \pi_{s}/C_{s}=0}=\frac{\tau_{s}}{F}$$

(15) $$H_{21}={\left(\frac{E}{\Delta \pi_{s}/C_{s}}\right)}_{I=0}=-\frac{\tau_{s}}{F}$$

(16) $$H_{22}={\left(\frac{E}{I}\right)}_{\Delta \pi_{s}/C_{s}=0}=\frac{1}{\kappa }$$

Equations (11) and (12) can also be written in a matrix form:

(17) $$\left[\begin{array}{c} \;J_{s}\\ E \end{array}\right]=\left[\begin{array}{cc} H_{11} & H_{12}\\ H_{21} & H_{22} \end{array}\right]\left[\begin{array}{c} \frac{\Delta \pi }{C_{s}}\\ I \end{array}\right]=\left[H\right]\left[\begin{array}{c} \frac{\Delta \pi }{C_{s}}\\ I \end{array}\right]$$

where $\left[H\right]$ is the hybrid matrix of Peusner coefficients $H_{ij}$ (*i*, *j* ∈ {1, 2}) for binary homogeneous electrolyte solutions. Equations (11)–(17) are among the H forms of the Kedem–Katchalsky equations.

Comparing the Equations (14) and (15), we can state that, for nondiagonal coefficients, the condition $H_{12}$ = $-H_{21}$ is fulfilled. For $J_{s}$ and E coupled to force $\Delta \pi_{s}/C_{s}$ and flux I, the relations $H_{11}H_{22}$ ≥ ${H_{12}}^{2}$ and $H_{11}H_{22}$ ≥ ${H_{21}}^{2}$ are valid. Furthermore, flux $J_{s}$ can only be coupled to the current density I if $H_{12}$ ≠ 0. In turn, flux I can only be coupled to the force $\Delta \pi_{s}/C_{s}$ if $H_{21}$ ≠ 0. Cross coefficients $H_{ij\left(i\neq j\right)}$ (*i*, *j* ∈ {1, 2}) describe the relationship between different irreversible processes.

(18) $$h_{12}=\frac{H_{12}}{\sqrt{H_{11}H_{22}}}=\frac{\tau_{s}}{F}{\left(\frac{\kappa }{C_{s}\omega_{s}}\right)}^{\frac{1}{2}}=-h_{21}$$

The expression formed from the coefficients $H_{ij}$ determines the degree of coupling between the observed processes (Kedem and Caplan coefficient) [9,10]. This means that the coefficient $h_{12}$ is a measure of the degree of coupling. If $h_{12}$ = 0, the irreversible processes are independent, while when $h_{12}$ = ±1, the irreversible processes are maximally coupled.

Using Peusner’s definition [9,10], the energy coupling parameter Q can be written as:

(19) $$Q_{H}=\frac{2\left|H_{12}H_{21}\right|}{4H_{11}H_{22}-2H_{12}H_{21}}=\frac{\left|h_{12}h_{21}\right|}{2-h_{12}h_{21}}=Q_{h}=\frac{\kappa {\tau_{s}}^{2}}{2C_{s}\omega_{s}F^{2}+\kappa {\tau_{s}}^{2}}$$

$Q_{H}$ can be used to analyze the efficiency of the biological and physico-chemical processes of energy conversion.

The next parameter determines the energy conversion efficiency $\;{\left(e_{H}\right)}_{max}$ and fulfills the condition 0 ≤ ${\left(e_{H}\right)}_{max}$ ≤ 1. This coefficient is determined by the equation:

(20) $${\left(e_{H}\right)}_{max}=\frac{\left|H_{12}H_{21}\right|}{H_{11}H_{22}{\left(1+\sqrt{1-\frac{H_{12}H_{21}}{H_{11}H_{22}}}\right)}^{2}}=\frac{2Q_{H}}{(1+Q_{H}){\left(1+\sqrt{1-\frac{2Q_{H}}{1+Q_{H}}}\right)}^{2}}=\frac{h_{12}h_{21}}{{\left(1+\sqrt{1-h_{12}h_{21}}\right)}^{2}}={\left(e_{h}\right)}_{max}$$

(21) $${\left(e_{H}\right)}_{max}=\frac{{\tau_{s}}^{2}\kappa }{2F^{2}C_{s}\omega_{s}-{\tau_{s}}^{2}\kappa +2\sqrt{F^{2}C_{s}\omega_{s}-{\tau_{s}}^{2}\kappa }}$$

Equations (20) and (21) describe the relationship between the degree of coupling and the efficiency of energy conversion. It is worth mentioning that full coupling ($h_{12}$ = 1) occurs at ${\left(e_{H}\right)}_{max}$ = 1. This means that the stationary states of membrane transport characterized by minimum entropy production are identical to the state with maximum efficiency.

### 2.3. Mathematical Model of Energy Conversion in the Membrane System

The dissipation function $\Phi_{S}$ is defined as product of absolute temperature (*T*) and entropy production ($d_{i}S/dt$) and can be used as the measure of *S*-energy dissipation. The mathematical equations for *S*-energy dissipation in a system with membrane separating homogeneous electrolytic solutions with different concentrations can be derived using the previously elaborated procedures [21].

The equation for the *H* version of the dissipation function for the membrane transport of electrolytic solutions in condition $J_{v}$ = 0 can be written as:

(22) $${\left(\Phi_{S}\right)}_{H}={\left(\Phi_{S}\right)}_{J_{s}}+{\left(\Phi_{S}\right)}_{I}=J_{s}\frac{\Delta \pi_{s}}{C_{s}}+IE$$

We will now calculate the ${\left(\Phi_{S}\right)}_{H}$ of Equation (23) using the H versions of the Kedem–Katchalsky–Peusner equations. Taking into account Equations (11) and (12), in Equation (22), we obtain:

(23) $${\left(\Phi_{S}\right)}_{H}=H_{11}{\left(\frac{\Delta \pi_{s}}{C_{s}}\right)}^{2}+\left(H_{12}+H_{21}\right)I\frac{\Delta \pi_{s}}{C_{s}}+H_{22}I^{2}$$

Because $H_{12}$ = $-H_{21},$ Equation (23) can be written as:

(24) $${\left(\Phi_{S}\right)}_{H}=H_{11}{\left(\frac{\Delta \pi_{s}}{C_{s}}\right)}^{2}+H_{22}I^{2}=C_{s}\omega_{s}{\left(\frac{\Delta \pi_{s}}{C_{s}}\right)}^{2}+\frac{1}{\kappa }I^{2}$$

The internal energy (U-energy) of membrane systems can be converted into free energy (F-energy) and dissipated energy (S-energy) [8]. The fluxes of these energies satisfy the following condition:

(25) $${\left(\Phi_{U}\right)}_{H}={\left(\Phi_{F}\right)}_{H}+{\left(\Phi_{S}\right)}_{H}$$

where ${\left(\Phi_{U}\right)}_{H}=A^{-1}dU/dt$ is the flux of *U*-energy, ${\left(\Phi_{F}\right)}_{H}=A^{-1}dF/dt$ is the flux of *F*-energy, ${\left(\Phi_{S}\right)}_{H}=TA^{-1}\;d_{i}S/dt$ is the flux of dissipated energy (*S*-energy), $d_{i}S/dt$ is the rate of entropy creation in the membrane system by irreversible processes (flux of cumulative entropy production), *T* is the absolute temperature, and *A* is the membrane surface area. Equations (23) and (24) show the H version of the S-energy dissipation. The ${\left(\Phi_{S}\right)}_{H}$ is the flux of dissipated energy, i.e., the time change of energy per unit area of the membrane expressed in W/m^2^. We can calculate the ${\left(\Phi_{F}\right)}_{H}$ and ${\left(\Phi_{U}\right)}_{H}$ for the homogeneous conditions using the following equation [8]:

(26) $${\left(e_{H}\right)}_{max}=\frac{{\left(\Phi_{F}\right)}_{H}}{{\left(\Phi_{U}\right)}_{H}}=\frac{{\left(\Phi_{F}\right)}_{H}}{{\left(\Phi_{F}\right)}_{H}+{\left(\Phi_{S}\right)}_{H}}$$

Transforming Equation (26), we get:

(27) $${\left(\Phi_{F}\right)}_{H}=\frac{{\left(e_{H}\right)}_{max}}{1-{\left(e_{H}\right)}_{max}}{\left(\Phi_{S}\right)}_{H}$$

(28) $${\left(\Phi_{U}\right)}_{H}=\frac{1}{1-{\left(e_{H}\right)}_{max}}{\left(\Phi_{S}\right)}_{H}$$

where ${\left(e_{H}\right)}_{max}$ is the energy conversion efficiency defined by means of Kedem–Caplan–Peusner coefficients.

In order for the denominator of Equations (27) and (28) to be different from zero, the condition ${\left(e_{H}\right)}_{max}$ ≠ 1 must be fulfilled. The internal energy (*U*-energy) of membrane systems can be converted into free energy (*F*-energy) and dissipated energy (*S*-energy) [8].

The ${\left(e_{H}\right)}_{max}$ coefficient is limited by the relation 0 ≤ ${\left(e_{H}\right)}_{max}$ ≤ 1; ${\left(e_{H}\right)}_{max}$ = 0 when $H_{12}H_{21}$ = 0 or $h_{12}h_{21}$ = 0, and ${\left(e_{H}\right)}_{max}$ = 1 when $H_{12}H_{21}$ = $H_{11}H_{22}$ and $h_{12}h_{21}$ = 1. Taking into account Equation (20), in Equation (27), we get:

(29) $${\left(\Phi_{F}\right)}_{H}=\frac{H_{12}H_{21}}{H_{22}{\left(1+\sqrt{1-\frac{H_{12}H_{21}}{H_{11}H_{22}}}\right)}^{2}-H_{12}H_{21}}{\left(\Phi_{S}\right)}_{H}$$

Taking into consideration Equation (20), in Equation (28), we get:

(30) $${\left(\Phi_{U}\right)}_{H}=\frac{H_{11}H_{22}H^{2}}{H_{11}H_{22}H^{2}-H_{12}H_{21}}{\left(\Phi_{S}\right)}_{H}$$

where:

(31) $$H=1+\sqrt{1-\frac{H_{12}H_{21}}{H_{11}H_{22}}}$$

Based on Equations (18)–(28), we can calculate the total internal *U*-energy and available *F*-energy, which is useful.

### 2.4. Evaluation of the Transport Properties of the Ultra-Flo 145 Dialyser Membranę

**Figure 3:**
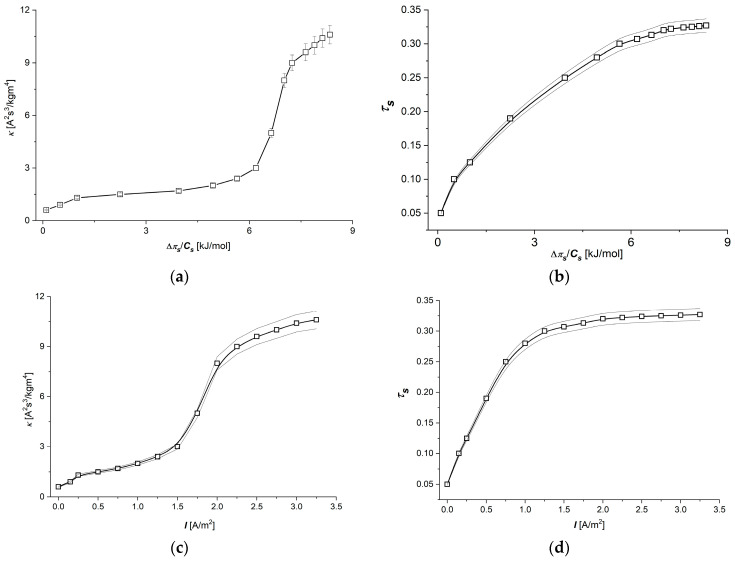
Dependencies $\kappa =f{\left(\Delta \pi /C_{s}\right)}_{I=1.5\;A/m^{2}}$ (**a**), $\tau_{s}=f{\left(\Delta \pi /C_{s}\right)}_{I=1.5\;A/m^{2}}$ (**b**), $\kappa =f{\left(I\right)}_{\Delta \pi /C_{s}=6.63\;\mathrm{kJ}/\mathrm{mol}\;}$ (**c**), and $\tau_{s}=f{\left(I\right)}_{\Delta \pi /C_{s}=6.63\;\mathrm{kJ}/\mathrm{mol}\;}$ (**d**) for Ultra Flo 145 Dialyzer membrane and aqueous NaCl solutions.

The transport coefficient $\omega_{s}$ of the Ultra Flo 145 Dialyser membrane appearing in Equation (3) in the studied range of NaCl concentrations is constant and is equal to $\omega_{s}$ = 5.5 × 10^−10^ mol/Ns. In turn, the values of transport coefficients κ and $\tau_{s}$ in the studied range of NaCl concentrations are concentration dependent. The dependencies $\kappa =f\left(\Delta \pi_{s}/C_{s},\;I=1.5\;A/m^{2}\right)$ and $\tau_{s}=f\left(\Delta \pi_{s}/C_{s},\;I=1.5\;A/m^{2}\right)$ are presented in Figure 3a,b, while Figure 3c,d show dependencies $\kappa =f\left(I,\;\Delta \pi_{s}/C_{s}=6.63\;\mathrm{kJ}/\mathrm{mol}\right)\;$ and $\tau_{s}=f(I,\;\Delta \pi_{s}/C_{s}=6.63\;\mathrm{kJ}/\mathrm{mol}$).

The electrical conductivity coefficient for the membrane (κ) is a parameter that is assumed to be constant in models for simple cases of membrane systems in which there are small thermodynamic forces. The use of larger thermodynamic forces, especially in systems with complex membrane structures, causes the approximation of constant coefficients to no longer be fulfilled. For this reason, models with descriptions that take into account the variability of coefficients are beginning to play an increasingly important role in the thermodynamics of non-equilibrium processes. As can be seen in Figure 3a,c, the electrical conductivity coefficient of the Ultra Flo 145 membrane depends on both $\Delta \pi_{s}/C_{s}$ and I. With the increase of both $\Delta \pi_{s}/C_{s}$ and I, the membrane conductivity for ions increases, while for lower values of control parameters ($\Delta \pi_{s}/C_{s}$ < 6 kJ/mol or I < 1.8 A/m^2^), the changes are small. Above these values, the rates of change of membrane electrical conductivity are significantly higher. At high values of control parameters (for $\Delta \pi_{s}/C_{s}$ > 8 kJ/mol or I > 2.3 A/m^2^), the rate of increase of the electrical conductivity value of the membrane is slower and decreases with the increase of the control parameter. These effects may be related to the interactions of ions with the membrane, with other ions and changes in the hydration shells of ions during their transport through the membrane. The visible increase in the rate of change of conductivity above a some value of the control parameter may be the result of a suitable increase in the concentration of transported ions in the membrane, which probably causes a facilitated flow of ions through the membrane, probably related to the effect of “screening” the interaction of ions with the membrane by other ions. When the next large threshold values of control parameters are exceeded, this effect weakens, which results in a decrease in the rate of electrical conductivity with an increase in the control parameter.

In turn, the ion transference number, whose dependence on the control parameters is presented in Figure 3b,d, may depend, similarly to electrical conductivity, on the density of the transferred ions in the membrane. In both cases, the ion transfer number increases with increasing control parameters, and the rate of the increase of this coefficient is increasingly slower. Probably, above sufficiently large values of the control parameters (e.g., for $\pi_{s}/C_{s}$ > 7.5 kJ/mol or I > 1.3 A/m^2^), a “saturation” effect can be observed, i.e., the ion transfer number becomes established for sufficiently large values of the control parameters.

These dependencies show that a nonlinear model connected with Equations (8) and (9) should be used for an accurate description of membrane processes, in our case, taken into account through the dependence of the transport coefficients of models *κ* and $\tau_{s}$ on the control parameters.

The *κ* and $\tau_{s}$ coefficients that determine the electrical transport properties of the membrane play an important role in ion transport through both artificial and biological membranes. As is shown in Figure 3, in the range of lower values of both control parameters (up to about 1 A/m^2^ or up to about 6 kJ/mol), the value of the coefficient $\tau_{s}$ of the Ultra Flo 145 Dialyser membrane changes only slightly, which, with some approximation, can be considered consistent with Kedem–Katchalsky–Peusner formalism for the area of linear dependence between control parameters.

However, for high values of $\Delta \pi /C_{s}$ and I, an observed decrease in the rate of change in ionic conductivity with an increase of one of the control parameters may indicate the observed tendency for the appearance of a “saturation effect” related to the limited capacity of the membrane to transport ions at high values of thermodynamic forces (this is particularly evident for electrical conditions). The shape of the curves for *κ* and $\tau_{s}$, which are easily measurable parameters characterizing the electrical properties of the membrane, are the starting point for obtaining further parameters in the presented model and provide more information about the phenomenon of electrolyte transport through the membrane.

## 3. Results and Discussion

### 3.1. The Characteristics $H_{ij}=f(\Delta \pi_{s}/C_{s}$ *,* I *)* *(i, j* *∈* *{1, 2})*

**Figure 4:**
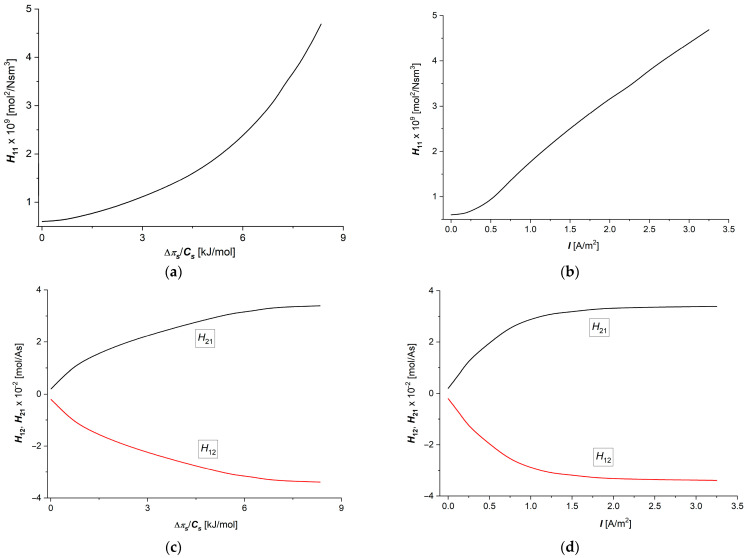
Graphic illustration of dependencies: (**a**) $H_{11}=f{\left(\Delta \pi /C_{s}\right)}_{I=1.5\;A/m^{2}}$, (**b**) $H_{11}=f{\left(I\right)}_{\Delta \pi /C_{s}=6.63\;\mathrm{kJ}/\mathrm{mol}\;}$, (**c**) $H_{12}=-H_{21}=f{\left(\Delta \pi_{s}/C_{s}\right)}_{I=1.5\;A/m^{2}}$, and (**d**) $H_{12}=-H_{21}=f{\left(I\right)}_{\Delta \pi /C_{s}=6.63\;\mathrm{kJ}/\mathrm{mol}\;}$ for aqueous NaCl solutions.

**Figure 5:**
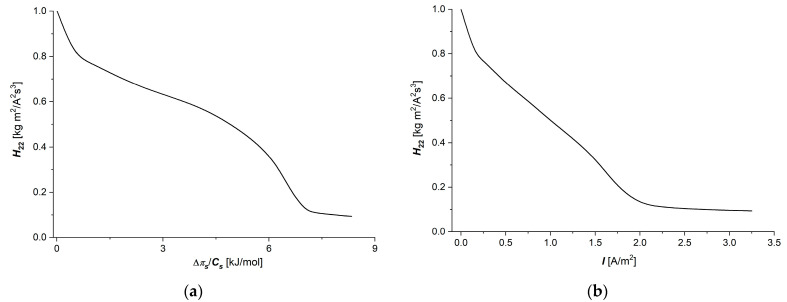
Graphic illustration of dependencies $H_{22}=f{\left(\Delta \pi /C_{s}\right)}_{I=1.5\;A/m^{2}}$ (**a**) and $H_{22}=f{\left(I\right)}_{\Delta \pi /C_{s}=6.63\;\mathrm{kJ}/\mathrm{mol}\;}$ (**b**) for aqueous NaCl solutions.

Calculations of the coefficients $H_{ij}=f\left(\Delta \pi_{s}/C_{s},\;I=1.5\;A/m^{2}\right)$ (i, j ∈ {1, 2}) were performed for the following data: R = 8.31 J/mol K, T = 295 K, F = 9.65 × 10^4^ C/mol, $C_{l}$ = 1 mol/m^3^, $C_{h}$ ∈ {1 ÷ 20 mol/m^3^} and $\Delta \pi_{s}/C_{s}$ ∈ {0 ÷ 8.34 kJ/mol}, and $z_{s}$ = 1. To calculate the dependencies $H_{ij}=f\left(\Delta \pi_{s}/C_{s},\;I=1.5\;A/m^{2}\right)$, $H_{ij}=f\left(I,\;\Delta \pi /C_{s}=6.63\;\mathrm{kJ}/\mathrm{mol}\;\right)$, and (i, j ∈ {1, 2}), Equations (13)–(16) and (18) were used. The results of the calculations are presented in Figure 4a–d and Figure 5a,b.

It can be seen in Figure 4a–d that the graphs illustrating the dependencies $H_{11}=f\left(\Delta \pi_{s}/C_{s},\;I=1.5\;A/m^{2}\right)$ and $H_{11}=f\left(I,\;\Delta \pi /C_{s}=6.63\;\mathrm{kJ}/\mathrm{mol}\;\right)$ are nonlinearly increasing functions of $\Delta \pi_{s}/C_{s}$ or I. Equation (13) shows that at a fixed value of $\omega_{s}$, the value of $H_{11}$ is a linear function of $C_{s}$. While the graph shown in Figure 4b approximately fulfills this criterion, the graph shown in Figure 4a shows significant deviations from linearity. These deviations can be explained using the equation for $H_{11}$, which can be obtained by including Equations (8)–(10) in Equation (13). After this operation, Equation (13) takes the form:

(32) $$H_{11}=\frac{{C_{s}}^{2}{\varphi_{w}}^{2}\vartheta }{C\Delta xf_{sw}^{0}}$$

This equation shows that at a fixed value of ϑ and Δx, the value of $H_{11}$ is directly pro-portional to ${C_{s}}^{2}{\varphi_{w}}^{2}$ and inversely proportional to $f_{sw}^{0}$. The curve shown in Figure 4a shows that the shape of this curve is determined by the ${C_{s}}^{2}{\varphi_{w}}^{2}$ factor. The curves illustrating the dependences $H_{12}=f\left(\Delta \pi_{s}/C_{s},\;I=1.5\;A/m^{2}\right)$ and $H_{12}=f\left(I,\;\Delta \pi /C_{s}=6.63\;\mathrm{kJ}/\mathrm{mol}\;\right)$ show that the values of $H_{12}$ coefficients increase nonlinearly with increasing values of $\Delta \pi_{s}/C_{s}$ or I. In contrast, the curves illustrating the dependencies $H_{21}=f\left(\Delta \pi_{s}/C_{s},\;I=1.5\;A/m^{2}\right)$ and $H_{21}=f\left(I,\;\Delta \pi /C_{s}=6.63\;\mathrm{kJ}/\mathrm{mol}\;\right)$ show that the value of $H_{21}$ coefficient decreases nonlinearly with increasing values of $\Delta \pi_{s}/C_{s}$ or I. Equation (14) shows that the value of $H_{12}$ is directly proportional to the $\tau_{s}$ coefficient, which is described by Equation (10). From this equation, it follows that the nature of the dependencies for $H_{12}$ and $H_{21}$ as functions of $\Delta \pi_{s}/C_{s}$ or I are determined by the curves for $\tau_{s}$.

Including Equation (10) in Equations (14) and (15), we get the following:

(33) $$H_{12}=\frac{1}{F}\frac{f_{1w}}{f_{2w}}{\left(\frac{C_{s}\varphi_{w}}{X}\right)}^{2}=-H_{21}$$

The dependencies $H_{22}=f\left(\Delta \pi_{s}/C_{s},\;I=1.5\;A/m^{2}\right)$ and $H_{22}=f\left(I,\;\Delta \pi /C_{s}=6.63\;\mathrm{kJ}/\mathrm{mol}\;\right)$ are shown in Figure 5a,b, suitable for aqueous NaCl solutions.

The curves shown in Figure 5a,b show that the value of the $H_{22}$ coefficient decreases nonlinearly with increasing values of $\Delta \pi_{s}/C_{s}$ or I. Equation (14) shows that the value of $H_{22}$ is inversely proportional to coefficient κ. This means that a hyperbola would be expected as a solution. Although the curves shown in Figure 5a,b demonstrate a decreasing trend, they are also characterized by significant deviations from the hyperbolic course. An explanation for this shape of the curves in these figures can be made using the equation obtained from Equations (9) and (16). This equation takes the form:

(34) $$H_{22}=\frac{f_{sw}^{0}}{\vartheta \;X\;F^{2}}$$

From this equation, it follows that at a fixed value of ϑ and Δx, the value of $H_{22}$ is directly proportional to $f_{sw}^{0}$ and inversely proportional to ion concentration in the membrane matrix (X). If X grows faster than $f_{sw}^{0}$, we get a hyperbola. Deviations from hyperbola are perhaps caused by the accumulation and/or depletion of ions in some sectors of the membrane structure.

### 3.2. Characteristics $h_{ij}=f\left(\Delta \pi_{s}/C_{s},I\right)$ *, (i, j* *∈* *{1, 2, 3}) and $Q_{H}=f\left(\Delta \pi_{s}/C_{s},I\right)$*

**Figure 6:**
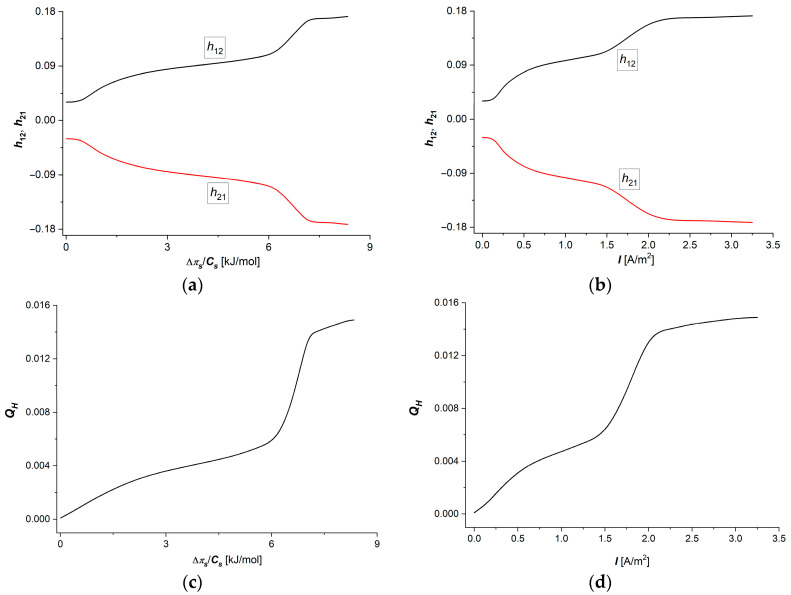
Graphic illustration of dependencies $h_{12}=-h_{12}=f{\left(\Delta \pi /C_{s}\right)}_{I=1.5\;A/m^{2}}$ (**a**), $h_{12}=-h_{12}=f{\left(I\right)}_{\Delta \pi /C_{s}=6.63\;\mathrm{kJ}/\mathrm{mol}\;}$ (**b**), $Q_{H}=f{\left(\Delta \pi /C_{s}\right)}_{I=1.5\;A/m^{2}}$ (**c**), and $Q_{H}=f{\left(I\right)}_{\Delta \pi /C_{s}=6.63\;\mathrm{kJ}/\mathrm{mol}\;}$ (**d**) for aqueous NaCl solutions.

Taking into account the dependencies $H_{ij}=f\left(\Delta \pi_{s}/C_{s},\;I=1.5\;A/m^{2}\right)$ and $H_{ij}=f(I,$ $\Delta \pi_{s}/C_{s}$ = 6.63 kJ/mol), (i, j ∈ {1, 2}) shown in Figure 6a–d, in Equation (20), the dependencies $h_{12}=f\left(\Delta \pi_{s}/C_{s},\;I=1.5\;A/m^{2}\right)$ and $h_{12}=f(I,$ $\Delta \pi_{s}/C_{s}$ = 6.63 kJ/mol) were calculated. The curves presented in Figure 6a,b show that the characteristics $h_{12}=f\left(\Delta \pi_{s}/C_{s},I=1.5\;A/m^{2}\right)$ and $h_{12}=f(I,$ $\Delta \pi_{s}/C_{s}$ = 6.63 kJ/mol) are nonlinear and $h_{12}$ = $-h_{21}$.

Considering Equations (8)–(10), in Equation (18), we obtain the “frictional” version of this equation:

(35) $$h_{12}=\frac{\tau_{s}}{F}{\left(\frac{\kappa }{C_{s}\omega_{s}}\right)}^{\frac{1}{2}}=\frac{f_{1w}C_{s}\varphi_{w}}{f_{2w}C}=-h_{21}$$

Considering the $h_{12}=f\left(\Delta \pi_{s}/C_{s},\;I=1.5\;A/m^{2}\right)$ and $h_{12}=f(I,$ $\Delta \pi_{s}/C_{s}$ = 6.63 kJ/mol) dependencies shown in Figure 6c,d and Equation (20), the dependencies $Q_{H}=f\left(\Delta \pi_{s}/C_{s},\;I=1.5\;A/m^{2}\right)$ and $Q_{H}=f(I,$ $\Delta \pi_{s}/C_{s}$ = 6.63 kJ/mol) were calculated. The curves presented in Figure 6c,d show that the characteristics $Q_{H}=f\left(\Delta \pi_{s}/C_{s},I=1.5\;A/m^{2}\right)$ and $Q_{H}=f(I,$ $\Delta \pi_{s}/C_{s}$ = 6.63 kJ/mol) are nonlinear, increasing functions of $\Delta \pi_{s}/C_{s}$ or I. Analysing the dependence of the energy coupling coefficient of membrane processes ($Q_{H}$), it is possible to find narrow range of changes in the control parameters (6 kJ/mol < $\Delta \pi_{s}/C_{s}<7\;\mathrm{kJ}/\mathrm{mol}\;$ or 1.4 A/m^2^ < I < 1.8 A/m^2^), in which the rate of change of $Q_{H}$ is much greater than in the other ranges of change in control parameters. This can be connected to the transition range of the control parameters, which separates low values of the control parameters with stable ion transport processes at low ion densities in the membrane from high values of the control parameters, for which high ion concentrations in the membrane contribute to the manifestation of the “saturation effect” in the membrane transport processes.

In Figure 6c,d, it can be seen that in the range of $\Delta \pi /C_{s}$ from 0 to 2.25 kJ/mol, the tangent of the angle of inclination of this section of the curve is $\Delta Q_{H}/\Delta \left(\pi_{s}/C_{s}\right)$ = 0.0014 mol/kJ, from 2.25 to 6.19 kJ/mol, $\Delta Q_{H}/\Delta \left(\pi_{s}/C_{s}\right)$ = 0.0007 mol/kJ, from 6.19 to 7.02 kJ/mol $\Delta Q_{H}/\Delta \left(\pi_{s}/C_{s}\right)$ = 0.009 mol/kJ, while from 7.24 to 8.34 kJ/mol = 0.001 mol/kJ. In the range from 0 to 0.75 A/m^2^, the tangent of the angle of inclination of this section of the curve is $\Delta Q_{H}/\Delta I$ = 0.005 m^2^/A from 0.75 to 1.5 A/m^2^, $\Delta Q_{H}/\Delta I$ = 0.003 m^2^/A, from 1.5 to 2 A/m^2^, $\Delta Q_{H}/\Delta I$ = 0.154 m^2^/A, while from 2 to 3.25 A/m^2^, $\Delta Q_{H}/\Delta I$ = 0.001 m^2^/A. Considering Equations (8)–(10), in Equation (19), we get the “frictional” version of the $Q_{H}$ coefficient:

(36) $$Q_{H}=Q_{h}=\frac{f_{1w}^{2}}{f_{1w}^{2}+2\;f_{2w}^{2}}$$

**Figure 7:**
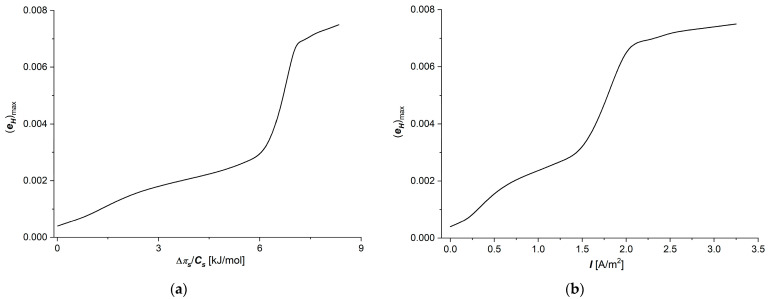
Graphic illustration of dependencies ($e_{H}{)}_{max}=f{\left(\Delta \pi /C_{s}\right)}_{I=1.5\;A/m^{2}}$ (**a**) and ($e_{H}{)}_{max}=f{\left(I\right)}_{\Delta \pi /C_{s}=6.63\;\mathrm{kJ}/\mathrm{mol}\;}$ (**b**) for aqueous NaCl solutions.

Considering the $h_{12}=f\left(\Delta \pi_{s}/C_{s},\;I=1.5\;A/m^{2}\right)$ and $h_{12}=f(I,$ $\Delta \pi_{s}/C_{s}$ = 6.63 kJ/mol) dependencies shown in Figure 7a,b and Equation (21), the dependencies ($e_{H}{)}_{max}=f\left(\Delta \pi_{s}/C_{s},\;I=1.5\;A/m^{2}\right)$ and ($e_{H}{)}_{max}=f(I,$ $\Delta \pi_{s}/C_{s}$ = 6.63 kJ/mol) were calculated. These dependencies are presented in Figure 7a,b. 

In Figure 7a,b, it can be seen that in the studied ranges of $\Delta \pi /C_{s}$ and *I*, there is the range of $\Delta \pi /C_{s}$ from 0 to 2.25 kJ/mol, where the tangent of the angle of inclination of this section of the curve is $\Delta {\left(e_{H}\right)}_{max}/\Delta \left(\pi_{s}/C_{s}\right)$ = 0.0007 mol/kJ, from 2.25 to 6.19 kJ/mol, $\Delta {\left(e_{H}\right)}_{max}/\Delta \left(\pi_{s}/C_{s}\right)$ = 0.00035 mol/kJ, from 6.19 to 7.02 kJ/mol $\Delta {\left(e_{H}\right)}_{max}/\Delta \left(\pi_{s}/C_{s}\right)$ = 0.0037 mol/kJ, while from 7.24 to 8.34 kJ/mol = 0.0027 mol/kJ. In the range of *I* from 0 to 0.75 A/m^2^, the tangent of the angle of inclination of this section of the curve is $\Delta {\left(e_{H}\right)}_{max}/\Delta \left(\pi_{s}/C_{s}\right)$ = 0.0028 m^2^/A from 0.75 to 1.5 A/m^2^, $\Delta {\left(e_{H}\right)}_{max}/\Delta I$ = 0.0025 m^2^/A, from 1.5 to 2 A/m^2^, $\Delta {\left(e_{H}\right)}_{max}/\Delta I$ = 0.0047 m^2^/A, while from 2 to 3.25 A/m^2^, $\Delta {\left(e_{H}\right)}_{max}/\Delta I$ = 0.003 m^2^/A. These ranges of change in the maximal energy conversion efficiency coefficient (${\left(e_{H}\right)}_{max}$) are similar to the ranges of change in the energy coupling coefficient of membrane processes ($Q_{H}$). Considering Equation (20), in Equation (35), we get the “frictional” version of the ${\left(e_{H}\right)}_{max}$ coefficient:

(37) $${\left(e_{H}\right)}_{max}=\frac{f_{1w}^{2}\;\sqrt{f_{1w}^{2}+f_{2w}^{2}}}{\left(f_{1w}^{2}+f_{2w}^{2}\right)\cdot \left(f_{2w}+\sqrt{f_{1w}^{2}+f_{2w}^{2}}\right)}$$

Figure 3a,c, Figure 6c,d and Figure 7a,b show that the shape of the curves illustrating the dependencies $\kappa =f\left(\Delta \pi_{s}/C_{s},\;I=1.5\;A/m^{2}\right)\;$(Figure 3a), $\kappa =f(I,\Delta \pi_{s}/C_{s}=6.63\;\mathrm{kJ}/\mathrm{mol}$) (Figure 3c), $Q_{H}=f\left(\Delta \pi_{s}/C_{s},\;I=1.5\;A/m^{2}\right)$ (Figure 6c), $Q_{H}=f(I,$ $\Delta \pi_{s}/C_{s}$ = 6.63 kJ/mol) (Figure 6d), ($e_{H}{)}_{max}=f\left(\Delta \pi_{s}/C_{s},\;I=1.5\;A/m^{2}\right)$ (Figure 7a), and ($e_{H}{)}_{max}=f(I,$ $\Delta \pi_{s}/C_{s}$ = 6.63 kJ/mol) (Figure 7b) are similar. In turn, Figure 3b,d and Figure 4c,d show that the shapes of the curves illustrating the dependencies $\tau_{s}=f\left(\Delta \pi_{s}/C_{s},\;I=1.5\;A/m^{2}\right)$ (Figure 3b), $\tau_{s}=f(I,\Delta \pi_{s}/C_{s}=6.63\;\mathrm{kJ}/\mathrm{mol}$ (Figure 3d), $H_{12}=f\left(\Delta \pi_{s}/C_{s},\;I=1.5\;A/m^{2}\right)$ (Figure 4c), and $H_{12}=f\left(I,\;\Delta \pi /C_{s}=6.63\;\mathrm{kJ}/\mathrm{mol}\;\right)$ are also similar.

### 3.3. The Characteristics ${\left[{\left(\Phi_{S}\right)}_{H}\right]}_{I=const}=f\left(\Delta \pi_{s}/C_{s}\right)$ *and* ${\left[{\left(\Phi_{S}\right)}_{H}\right]}_{\Delta \pi_{s}/C_{s}=const}=f\left(I\right)$

**Figure 8:**
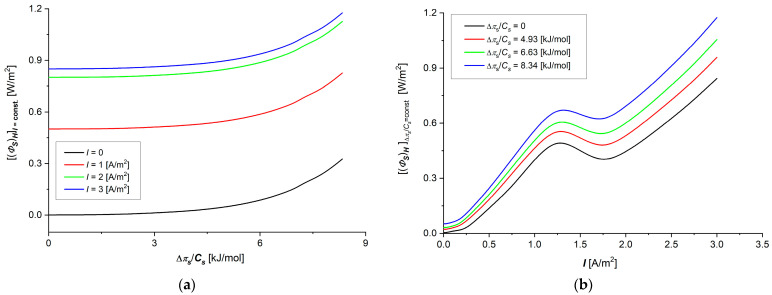
Graphic illustration of dependencies ${\left[{\left(\Phi_{S}\right)}_{H}\right]}_{I=const}=f\left(\Delta \pi_{s}/C_{s}\right)$ (**a**) and ${\left[{\left(\Phi_{S}\right)}_{H}\right]}_{\Delta \pi_{s}/C_{s}=const}=f\left(I\right)$ (**b**) for aqueous NaCl solutions.

Taking into account $H_{ij}=f\left(\Delta \pi_{s}/C_{s}\right)$, (i, j ∈ {1, 2}), shown in Figure 4a–d and Figure 5a,b, in Equation (23), the dependencies ${\left(\Phi_{S}\right)}_{H}=f\left(\Delta \pi_{s}/C_{s},\;I=\mathrm{const}.\right)$ and ${\left(\Phi_{S}\right)}_{H}=f(I,$ $\Delta \pi_{s}/C_{s}$ = const) were calculated. The results of calculations are presented in Figure 8a,b. The graphs shown in these figures are nonlinear, increasing the functions of control parameters $\Delta \pi_{s}/C_{s}$ (Figure 8a) and I (Figure 8b). In Figure 8a,b, it can be seen that ${\left(\Phi_{S}\right)}_{H}$ increases both with the increase of $\Delta \pi_{s}/C_{s}$ at a fixed value of I and with the increase of I at a fixed value of $\Delta \pi_{s}/C_{s}$. Taking into account Equations (13) and (16), in Equation (24), we get the frictional version of Equation (24):


(38) $${\left(\Phi_{S}\right)}_{H}=\frac{{C_{s}}^{2}{\varphi_{w}}^{2}\vartheta }{X\;\Delta x\;f_{iw}^{0}}{\left(\frac{\Delta \pi_{s}}{C_{s}}\right)}^{2}+\frac{f_{iw}^{0}}{\vartheta \;X\;F^{2}}I^{2}$$

The results of the calculations show that the value of the first component of Equation (38) is always greater than the value of the second component of this equation.

The flux of dissipated energy in membrane processes, presented in Figure 8a,b, depends on the control parameters and, as could be expected, is an increasing function of these parameters. The dependence ${\left[{\left(\Phi_{S}\right)}_{H}\right]}_{I=const}=f\left(\Delta \pi_{s}/C_{s}\right)$ is a nonlinear increasing function, where for small values of $\Delta \pi_{s}/C_{s}$, lower than 6 kJ/mol, the rate of change of the dissipated energy is smaller than in the range $\Delta \pi_{s}/C_{s}$ > 6 kJ/mol. We observe that the rate of increase of ${\left[{\left(\Phi_{S}\right)}_{H}\right]}_{I=const}$ increases with the increase of $\Delta \pi_{s}/C_{s}$. In turn, increasing the current density to a value of about 1.2 A/m^2^ in the membrane system causes an increase in the energy dissipated in membrane processes, after which, passing through a local maximum, the amount of dissipated energy decreases slightly, and then, current densities of greater than about 1.9 A/m^2^ increase again, with a similar rate of increase as low current density values. The relative changes in the flux of dissipated energy in the observed ranges of control parameters are greater for the current density changes than for the $\Delta \pi_{s}/C_{s}$ changes.

### 3.4. Characteristics ${\left[{\left(\Phi_{F}\right)}_{H}\right]}_{I=const}=f\left(\Delta \pi_{s}/C_{s}\right)$ and ${\left[{\left(\Phi_{F}\right)}_{H}\right]}_{\Delta \pi_{s}/C_{s}=const}=f\left(I\right)$ and ${\left[{\left(\Phi_{U}\right)}_{H}\right]}_{I=const}=f\left(\Delta \pi_{s}/C_{s}\right)$ and ${\left[{\left(\Phi_{U}\right)}_{H}\right]}_{\Delta \pi_{s}/C_{s}=const}=f\left(I\right)$

**Figure 9:**
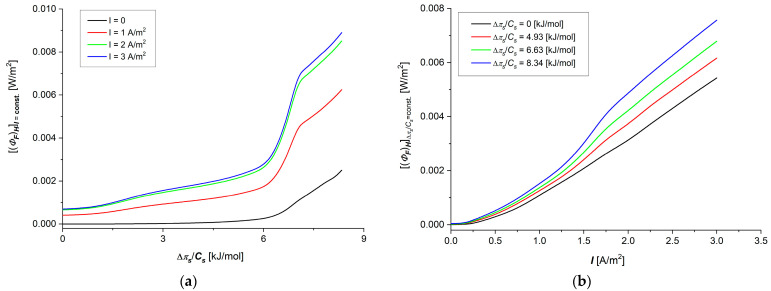
Graphic illustration of dependencies ${\left({\left[{\left(\Phi_{F}\right)}_{H}\right]}_{I=const}\right)}_{I=const}=f\left(\Delta \pi_{s}/C_{s}\right)\;$(**a**) and ${\left(\left[{\left(\Phi_{F}\right)}_{H}\right]\right)}_{\Delta \pi_{s}/C_{s}=const}=f\left(I\right)$ (**b**) for aqueous NaCl solutions.

Taking into account ($e_{H}{)}_{max}=f\left(\Delta \pi_{s}/C_{s}\right),$ shown in Figure 7a,b, in Equations (21) and (27), and dependencies ${\left[{\left(\Phi_{S}\right)}_{H}\right]}_{I=const}=f\left(\Delta \pi_{s}/C_{s}\right)$ and ${\left[{\left(\Phi_{S}\right)}_{H}\right]}_{\Delta \pi_{s}/C_{s}=const}=f\left(I\right)$, presented in Figure 8a,b, the dependencies ${\left[{\left(\Phi_{F}\right)}_{H}\right]}_{I=const}=f\left(\Delta \pi_{s}/C_{s}\right)$ and ${\left[{\left(\Phi_{F}\right)}_{H}\right]}_{\Delta \pi_{s}/C_{s}=const}=f\left(I\right)$ were calculated. These dependencies are shown in Figure 9a,b.

Comparing the data for ${\left[{\left(\Phi_{F}\right)}_{H}\right]}_{I=const.}=f\left(\Delta \pi_{s}/C_{s}\right)$ and ${\left[{\left(\Phi_{S}\right)}_{H}\right]}_{I=const.}=f\left(\Delta \pi_{s}/C_{s}\right),$ shown in Figure 8a,b, and the data for ${\left[{\left(\Phi_{F}\right)}_{H}\right]}_{\Delta \pi_{s}/C_{s}=const.}=f\left(I\right)$ and ${\left[{\left(\Phi_{S}\right)}_{H}\right]}_{\Delta \pi_{s}/C_{s}=const.}=f\left(I\right),$ shown in Figure 9a,b, it can be seen that ${\left[{\left(\Phi_{F}\right)}_{H}\right]}_{I=const.}$ ≈ 0.01${\left[{\left(\Phi_{S}\right)}_{H}\right]}_{I=const.}$ and ${\left[{\left(\Phi_{F}\right)}_{H}\right]}_{\Delta \pi_{s}/C_{s}=const.}$ ≈ 0.01${\left[{\left(\Phi_{S}\right)}_{H}\right]}_{\Delta \pi_{s}/C_{s}=const.}$.

The flux of useful energy in comparison with the flux of dissipated energy in the same conditions of measurement is much lower. This means that almost the entire flux of internal energy of the system associated with membrane processes is converted into an energy flux dissipated in the system. The useful energy flux also depends on both control parameters, and these dependencies are increasing and non-linear functions. If the useful energy flux increases evenly in almost the entire range of current densities (for I > 0.025 A/m^2^), then for the dependence of the useful energy flux on $\Delta \pi_{s}/C_{s}$, relatively small changes in this flux for small values of $\Delta \pi_{s}/C_{s}$ < 6 kJ/mol are followed by a rapid increase in the useful energy flux with the change of $\Delta \pi_{s}/C_{s}$.

## 4. Conclusions

All calculated coefficients of coupling between thermodynamic forces and fluxes in the H version of PNT ($H_{ij},\;i,\;j\;\in \;\left\{1,\;2\right\}$) depend nonlinearly on both control parameters $\Delta \pi_{s}/C_{s}$ and I. The coefficients $H_{11}$, $H_{12}$, and $H_{22}$ are positive over the entire range of the used NaCl concentrations. A positive value of the $H_{ij}$ coefficient means that an increase in the *j*-th stimulus causes an increase in the corresponding *i*-th flux. Nonlinear changes in the coefficient $H_{ij}$ make the force–flux relationship more complex. The greater the slope of the characteristics $H_{ij}=f\left(\Delta \pi_{s}/C_{s},I=const.\right)$, and $H_{ij}=f(I,$ $\Delta \pi_{s}/C_{s}=const.$) (i, j ∈ {1, 2}), the greater the nonlinear effect between thermodynamic forces and fluxes.

The nonlinearity of these characteristics is connected with the structure of the membrane and its frictional interactions with the individual transported substances and, thus, indirectly to the interaction between the transported substances in the membrane. The coupling coefficients $h_{ij}$ between the various processes in the membrane take values ranging from zero (no coupling) to one (full coupling). As can be seen in the calculations, an increase in I or $\Delta \pi_{s}/C_{s}$ causes an increase in coupling between ion transport processes in the Ultra Flo 145 Dialyser membrane. In addition, an increase in I or $\Delta \pi_{s}/C_{s}$ causes an increase in the coefficient of energy conversion efficiency ${\left(e_{H}\right)}_{max}$, as well as fluxes of free energy and dissipated energy for the Ultra Flo 145 Dialyser membrane during ion transport through the membrane.

The membrane transport of ions through the membrane Ultra Flo 145 dialyzer requires an extension of the linear model, for example, by making the model’s transport coefficients dependent on thermodynamic parameters.

## Data Availability

The raw data supporting the conclusions of this article will be made available by the authors on request.
